# Cosmetics of Castration

**Published:** 1897-02

**Authors:** 


### COSMETICS OF CASTRATION.

The esthetically inclined editor of the *Medical Record* suggested some time ago that the unsightly void left by orcheotomy should be filled by a celluloid testicle for cosmetic reasons. This suggestion (from a recent report of a discussion before the Chicago Medico-Legal Society) would seem to have fallen on fruitful soil. Discussing the question of castration of criminals, Dr. Gertrude G. Wellington remarked that a man castrated for disease wished something to take the place of the organ removed that would give him semblance of manhood. The only thing that could be found were some balls of celluloid, but they each contained a little bell. She would advise that in habitual criminals and sexual perverts after castration, these celluloid balls, with their jingling bells be inserted, so that as the man went about among women of the world the bells would proclaim him incapacitated.—*Medical Standard.*

